# Characterization of poplar GrxS14 in different structural forms

**DOI:** 10.1007/s13238-014-0042-3

**Published:** 2014-03-18

**Authors:** Lei Wang, Yifei Li, Jean-Pierre Jacquot, Nicolas Rouhier, Bin Xia

**Affiliations:** 1 Beijing Nuclear Magnetic Resonance Center, Peking University, Beijing, 100871 China; 2 College of Chemistry and Molecular Engineering, Peking University, Beijing, 100871 China; 3 College of Life Science, Peking University, Beijing, 100871 China; 4 Unité Mixte de Recherches INRA UHP 1136, Interaction Arbres Microorganismes, IFR 110 Genomique, Ecophysiologie et Ecologie Fonctionnelles Université Henri Poincaré, BP 239, 54506 Vandoeuvre Cedex, France

**Dear Editor,**

Glutaredoxins (Grxs) are glutathione-dependent thiol disulfide oxidoreductases of the thioredoxin family present in all organisms from bacteria to human (Noguera et al., 2005). Depending on their active site sequence, Grxs are essentially classified into three families: the dithiol Grxs, the monothiol Grxs and the CC type restricted to plants (Rouhier et al., 2008). Grxs play important biological functions in plants, such as oxidative stress responses, iron-sulfur (FeS) cluster assembly, and cell signaling, etc. (Rouhier et al., 2008). There are totally 31 Grxs isoforms in *Arabidopsis thaliana*, and 19 Grx isoforms in *Populus trichocarpa* (Rouhier et al., 2004). For Grxs of *Populus trichocarpa*, structures of GrxC1, GrxC4 and GrxS12 have been resolved, all belong to dithiol Grxs (Noguera et al., 2005; Feng et al., 2006; Rouhier et al., 2007; Couturier et al., 2009). The only structure available for monothiol Grxs in plants is that of *Arabidopsis* Grxcp, which is also called GrxS14 or CAXIP1 (Cheng and Hirschi, 2003; Li et al., 2010).

It was found that *Arabidopsis* GrxS14 is a new class of signaling molecules in plants that can regulate the Ca^2+^ transport activity of CAX1 (cation exchangers) by interacting with the N-terminal region of CAX1 (Cheng and Hirschi, 2003). It was suggested that *Arabidopsis* GrxS14 functions to protecting cells against protein oxidative damage (Cheng et al., 2006). Both *Arabidopsis* and poplar GrxS14 are monothiol Grxs located in the chloroplasts, which exist as an apo form and a holo form bridged by a [2Fe-2S] cluster with two external glutathione (GSH) ligands, and they can complement a yeast *grx5* mutant defective in FeS cluster assembly *in vivo* (Bandyopadhyay et al., 2008). It was proposed that *Arabidopsis* and poplar GrxS14 may function as scaffold protein for the assembly of [2Fe-2S] cluster, as GrxS14 can transfer intact cluster to physiologically relevant acceptor proteins which is regulated by GSH (Bandyopadhyay et al., 2008; Wang et al., 2012; Liu et al., 2013).

Here we report the solution structure of reduced poplar GrxS14 and structure models for the non-covalent apo GrxS14 dimer and GrxS14/GSH complex, as well as the NMR characterization of holo GrxS14.

The quality of the 2D ^1^H-^15^N HSQC spectrum of apo GrxS14 at 1 mmol/L concentration was very poor (Fig. S1), and very few signals could be observed in 3D triple-resonance NMR spectra. Dilution of the sample did not improve the quality of NMR spectra very significantly. Interestingly, much better NMR spectra were obtained with the addition of GSH (Fig. S1). Although apo GrxS14 appeared to be a monomer on the gel filtration column, analytical ultracentrifugation analysis showed two peaks (with molecular weight about 24 kDa and 12 kDa) for apo GrxS14 without GSH, while there was only one peak at ~12 kDa for apo GrxS14 with GSH (Fig. S2A and S2B). All these suggest that apo GrxS14 should be in a monomer-to-dimer equilibrium, and the dimerization can be inhibited by GSH. Thus, there exists another type of GrxS14 dimer in addition to the holo GrxS14 dimer assembled with a [2Fe-2S] cluster. Based on the analytical ultracentrifugation data (Fig. S2A), the dissociation constant of GrxS14 monomer-to-dimer equilibrium is estimated to be ~0.4 mmol/L.

We determined the solution structure of reduced monomeric GrxS14 using NMR data collected on protein samples in the presence of 20 mmol/L GSH. A summary of structural restraints used in the structure calculation and statistics for the structure ensemble is listed in Table S1. Residues 5–109 of apo GrxS14 form a compact thioredoxin fold structure while the first four residues are flexible (Fig. 1A). It comprises five α-helices and four β-strands, the four β-strands constitute a mixed β-sheet as the core of structure (Fig. 1B). Helices α1 and α3 are packed on one side of the β-sheet, while α2, α4 and α5 are on the other side.

**Figure 1:**
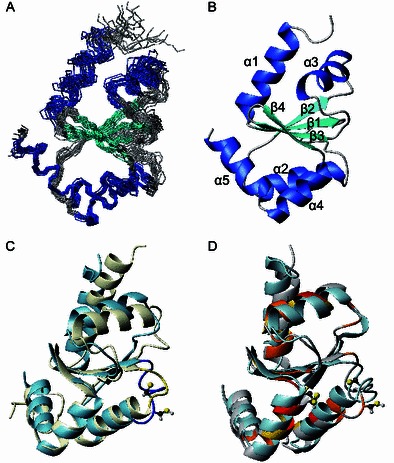
**Solution structure and structural comparison of reduced apo GrxS14**. (A) The backbone superimposition of 20 structures. (B) The ribbon representation of the mean structure. (C) Structural comparison of poplar GrxS14 (sky blue) and poplar GrxC1 (light yellow) (PDB 1Z7P, 1Z7R). Loop regions between β1 and α2 are shown as blue and yellow, respectively. (D) Structural comparison of poplar GrxS14 (light gray) and *Arabidopsis* GrxS14 (light blue) (PDB 3IPZ). Residues with significant combined NH chemical shift changes upon GSH binding are indicated on the structure of poplar GrxS14 by yellow color (0.05 < δ < 0.1 ppm), orange color (0.1 ≤ δ < 0.2 ppm), and orange red (δ ≥ 0.2 ppm), respectively. The side-chains of conserved active site cysteine are illustrated as ball and stick

The overall fold of poplar GrxS14 is similar to other Grxs. The RMSD of backbone heavy atoms in secondary structure regions is 1.7 Å between poplar and *Arabidopsis* GrxS14 which shares an 80% sequence identity (Figs. 1D and S3A). The relatively large RMSD between the two may be due to that the poplar apo GrxS14 was determined in the presence of GSH, while no GSH is in the crystal structure of *Arabidopsis* GrxS14. When comparing to dithiol Grxs, the major difference is at the loop region between β1 and α2: poplar GrxS14 contains ten amino acid residues, while only four residues in poplar GrxC1 and human Grx2 (Fig. 1C). Sequence alignment indicated that monothiol Grxs all possess a long loop in this region, whereas the dithiol Grxs usually have a short loop (Fig. S3). This long loop before the active site (CGFS) is a structural characteristic of monothiol Grxs.

The dimerization of apo GrxS14 was investigated using NMR spectroscopy. Comparison of 2D ^1^H-^15^N HSQC spectra of apo GrxS14 at different concentrations revealed that residues with significant concentration-dependent NH chemical shift changes are F35, Q37, K66, W71, G86, D88, I89, V91, E92 and S96, and these residues should be involved in the dimer interface (Figs. 2A and S4). Based on the structure models of apo GrxS14 dimer calculated using HADDOCK 2.0 (supplementary methods), the dimer interface is located at helices α2, α3 and α4, and loops β1-α2, α3-β3 and β4-α4 (Fig. 2B). In the dimer interface, there should be aromatic stacking interaction between the two phenyl groups of F35 since the two rings are close, and side-chains of K66 from one molecule and D88 or E92 from the other are in a distance to form salt bridges (~2 Å) (Fig. 2B).

**Figure 2:**
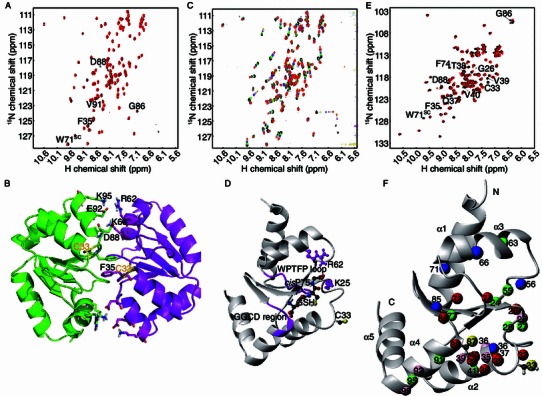
**Structural characterization of apo GrxS14 dimer, GrxS14/GSH complex, and holo GrxS14**. (A) Superimposed 2D ^1^H-^15^N HSQC spectra of apo GrxS14 at 0.1 mmol/L (black) and 0.4 mmol/L (red). (B) A structure model of apo GrxS14 dimer (in green and magenta). Side-chains of some interface residues are shown. Side-chains of the active site C33 residues are shown as yellow. (C) 2D ^1^H-^15^N HSQC spectra of GSH titration of apo GrxS14 (0.1 mmol/L). The colors represent different [GSH]:[GrxS14] ratios from 0 (black) to 230:1 (red). (D) Structure models of GrxS14/GSH complex. GSH is shown as ball and stick. The GSH binding site of GrxS14 is labeled with residues number and colored in magenta. The side-chains of K25 and R62 are also shown in magenta. The active site C33 is also indicated. (E) Overlay of 2D ^1^H-^15^N HSQC spectra of holo (red) and apo (black) poplar GrxS14. Only the assignments of residues with missing NH signals in holo protein are labeled. (F) Mapping of residues with large chemical shift changes or peak intensity changes. Nitrogen atoms corresponding to missing NH peaks in holo GrxS14 are shown as red spheres, those with large intensity reduction are shown as green spheres, and those with large chemical shift changes are shown as blue spheres. The C^α^ atoms of residues with large chemical shift changes are shown as pink spheres. The sulfur atoms of C33 and C87 are shown as yellow spheres

To verify these interactions, two mutants F35A and D88A/E92A of GrxS14 were generated. Analytical ultracentrifugation analysis revealed that the amount of dimer fraction is significantly reduced for the two mutants without GSH (Figs. S2C and S2D), indicating that the aromatic stacking and the salt bridges are critical for the dimerization. Thus, the docking model of the GrxS14 dimer is valid.

While most Grxs are found to be monomeric protein (Lillig et al., 2008), it was reported that the reduced poplar GrxC4 can self-associate into dimers with *K*_d_ in mmol/L range (Noguera et al., 2005). Comparing the dimer interface between GrxS14 and GrxC4, the residues involved aromatic stacking (F35 in GrxS14 and Y29 in GrxC4) and electrostatic interactions (D88, E92 in GrxS14 and D85, E89 in GrxC4) are conserved in sequences (Fig. S3B), and the dimer interface of GrxS14 should be similar to that of GrxC4 (Noguera et al., 2005). Although there is only one molecule in the crystallographic asymmetric unit for the crystal structure of *Arabidopsis* GrxS14 (Li et al., 2010), we found that a similar dimer interface exists between two molecules in two asymmetric units (Fig. S5B). The dimer interface of *Arabidopsis* GrxS14 is mainly involved in aromatic contact between two F99 residue and electrostatic interaction between K130 and E156, similar to those in poplar apo GrxS14 dimer (Fig. S5). However, it is expected that there are differences for the details of the two dimer interfaces, since our poplar apo GrxS14 dimer structure is a docking model and the dimer interface for *Arabidopsis* GrxS14 may be distorted due to crystal packing.

We have also performed NMR titration experiments to monitor the interaction between apo GrxS14 and GSH. The perturbation of GSH on NH signals of apo GrxS14 is rather dramatic, as residues with significant combined NH chemical shift changes (>0.05 ppm) between free protein and that with 230-fold of GSH are distributed on all secondary structure elements (Figs. 2C and S6). This is consistent with the above mentioned relatively large RMSD between *Arabidopsis* GrxS14 and poplar apo GrxS14, as our poplar apo GrxS14 structure is determined in the presence of GSH (Fig. 1D). This may suggest that GSH binding can trigger relatively global conformation adjustment for poplar apo GrxS14.

NH signals from residues at different areas were chosen to fit the dissociation constant and similar *K*_d_ values (apparent *K*_d_ ~5 mmol/L) were obtained, which suggests that only one GSH binds GrxS14 (Fig. S7). Based on the chemical shift perturbation of GSH, we calculated structure models of GSH bound GrxS14 using HADDOCK 2.0 (supplementary methods), which reveal that GSH does bind at the conserved GSH binding motif of Grxs (Lillig et al., 2008). In the models, GSH mainly contacts three regions of GrxS14 (Fig. 2D): loop β1-α2 and α3 (K25 and R62) stabilize the C-terminus of GSH through hydrogen bonds and electrostatic interactions; the GGCD region (between β4 and α4) stabilizes the N-terminus of GSH through hydrogen bond and dipolar interaction (α4 helix); and the WPTFP loop (between α3 and β3) is well conserved in Grxs, especially the *cis* configuration of P75, should provide enhanced favorable contacts with GSH (Couturier et al., 2009). The structural model for GrxS14/GSH complex may provide a recognition mechanism for Grxs to target glutathionylated protein substrates in the reversible protein glutathionylation process. In addition, as the GSH binding site was included in the dimer interface of apo GrxS14 (Figs. 2B,2D and S8C), the binding of GSH is incompatible with the dimerization of GrxS14, which explains why GSH can prevent the dimerization of GrxS14 (Fig. S2B).

Grx structures with GSH covalently linked to the active site cysteine by disulfide have been reported for *E. coli* Grx3 (PDB 3GRX), human Grx1 (PDB 1B4Q), yeast Grx1 (PDB 3C1R), *Arabidopsis* GrxC5 (PDB 3RHB) and poplar GrxS12 (PDB 3FZ9). Grx structures with non-covalently bound GSH are also available for human Grx2 (PDB 2FLS), poplar GrxC4 (Noguera et al., 2005) and yeast Grx6 (PDB 3L4N), all belong to the classical dithiol Grxs. It is found that the GSH binding modes are quite similar at the conserved GSH binding motif (three regions mentioned above) for both monothiol and dithiol Grxs, whether GSH bound non-covalently or covalently with mixed disulfide (Figs. S8A and S8B). Meanwhile, ligand GSHs in holo Grxs also show very similar binding modes at the conserved GSH binding motif (Fig. S8B).

Furthermore, we have characterized the holo GrxS14 with NMR spectroscopy. The backbone resonance assignments of holo GrxS14 were determined for ~90% of the total residues (Wang et al., 2011). A comparison of the 2D ^1^H-^15^N HSQC spectra of apo and holo GrxS14 is shown in Fig. 2E. NH peaks of residues G26, C33, F35, Q37, T38, V39, V40, W71 (side chain NH), F74, G86 and D88 are missing (Fig. 2E). Most of ^13^C^α^ chemical shifts are obtained for holo GrxS14, except for residues P31, C33, G34, T73 and G86. On the other hand, only 5 residues have significant NH chemical shift changes (*δ* > 0.05 ppm) among the 86 assigned NH signals: S36, L56, K66, W71, G85 and V91 (Figs. 2F and S9A). NH signals of residues M24, T27, K28, Q41, I55, Q63, L77, V91 and K95 show significantly intensity reduction compared to the apo form (Figs. 2F and S9B). The ^13^C^α^ chemical shift changes are quite small for most residues except for G26, F35, S36, V39, E92 and K95 (*δ*_Cα_ > 0.3 ppm) (Figs. 2F and S9C).

Most of missing and weaker NH signals should be due to the paramagnetic effect of the [2Fe-2S] cluster, as they are from residues located around the CGFS active site and/or the GSH binding site, where the [2Fe-2S] cluster is presumably coordinated by the active site cysteines and the two GSH cysteines (Fig. 2F). The paramagnetism of the [2Fe-2S] cluster could cause NMR signals broadened and/or hyperfine-shifted for residues over 10 Å away from the cluster, dependent on the magnetic susceptibility tensor of the cluster (Feng et al., 2006). Thus, although some of the residues are not very close to the active site, their NH signals are also affected. Since most NH peaks in the 2D ^1^H-^15^N HSQC spectrum of holo GrxS14 can be superimposed on those of apo GrxS14, and the residues without significant ^13^C^α^ chemical shift changes are distributed in all secondary structure elements (Fig. 2E and 2F), the structure of each subunit in the holo GrxS14 should largely remain the same as the apo protein.

As a summary, we have determined the solution structure of apo GrxS14, and investigated the structures of the apo GrxS14 dimer, its complex with GSH, and holo GrxS14. The conserved GSH binding site in apo GrxS14 implies the recognition mechanism of monothiol Grxs to various glutathionylated proteins as substrates. While GSH serves as FeS cluster ligand in holo GrxS14, GSH also inhibits the non-covalent dimerization of apo GrxS14. As the *K*_d_ values of GSH binding is in mmol/L level and the concentration of GSH is fluctuating in the mmol/L range in plant chloroplast (Rouhier et al., 2008), GSH may have a regulation effect on the dimerization of GrxS14 *in vivo.* Therefore, it seems that GSH may play a more complicated role in the structure and function of GrxS14, as we have previously reported that GSH can regulate the FeS cluster transfer from holo GrxS14 to apo ferredoxin (Wang et al., 2012). Further studies are needed to uncover the hidden physiological roles of GSH on GrxS14.

## Electronic supplementary material

Below is the link to the electronic supplementary material.

Supplementary material 1 (PDF 745 kb)
