# Supplementary material for: Characterization of poplar GrxS14 in different structural forms
Source: Protein Cell. 2014 Mar 18;5(5):329–33. doi: 10.1007/s13238-014-0042-3 (PMC3996159; doi:10.1007/s13238-014-0042-3)
Supplement: Supplementary file 1 — Supplementary material 1 (PDF 745 kb) [file 13238_2014_42_MOESM1_ESM.pdf]

SUPPLEMENTARY MATERIALS

Characterization of poplar GrxS14 in different structural forms

Running Title: NMR studies of poplar GrxS14

KEY WORDS: glutaredoxin, glutathione, solution structure, dimerization, GSH binding site, NMR.

Lei Wang[1,2], Yifei Li[1], Jean-Pierre Jacquot[4], Nicolas Rouhier[4], and Bin Xia[1,2,3,*]

[1] Beijing Nuclear Magnetic Resonance Center, Peking University, Beijing, 100871, P.R. China

[2] College of Chemistry and Molecular Engineering, Peking University, Beijing, 100871, P.R. China

[3] College of Life Science, Peking University, Beijing, 100871, P.R. China

[4] Unité Mixte de Recherches INRA UHP 1136, Interaction Arbres Microorganismes, IFR 110 Genomique, Ecophysiologie et Ecologie Fonctionnelles Université Henri Poincaré, BP 239, 54506, Vandoeuvre Cedex, France

*Correspondence: binxia@pku.edu.cn

NMR sample preparation

The apo and holo protein were purified according to the procedure described previously (Wang et al., 2011). The NMR samples contain 0.2 mM (apo form), 0.5 mM (holo form) uniformly ^{15}N- and/or ^{13}C-labeled protein in 30 mM Tris-HCl buffer with 50 mM NaCl and 95% H_2O/5% D_2O at pH 7.5, along with 0.01% NaN_3 and 0.01% DSS. All NMR samples had 20 mM GSH for both apo and holo proteins, and oxygen was removed from the samples in order to keep a reducing condition for stabilizing the samples.

Analytical ultracentrifuge experiment

The Sedimentation velocity experiments were carried out with a Beckman Coulter ProteomeLab^{TM} XL-I instrument. All AUC runs were carried out at the rotation speed of 60,000 rpm at 16 °C. The sample volume was 400 μL and the protein concentration was ~ 0.075 mM. A wavelength of 280 nm was used to record the UV absorption of the cells which scanned every minute for 5 h. The AUC data were analyzed using SEDFIT program (Schuck, 2000).

NMR spectroscopy

NMR experiments were performed at 25 ^{o}C on a Bruker Avance 500 MHz or an 800 MHz spectrometer with a cryoprobe. Proton chemical shifts were referenced to internal DSS. ^{15}N and ^{13}C chemical shifts were referenced indirectly to DSS (Markley et al., 1998). Three-dimensional ^{15}N-edited NOESY-HSQC and ^{13}C-edited aliphatic

and aromatic NOESY-HSQC spectra were collected with mixing times of 120 and 90 ms for the apo protein, respectively (Ferentz and Wagner, 2000). All NMR spectra were processed using NMRPipe (Delaglio et al., 1995) and analyzed with NMRView (Johnson and Blevins, 1994).

Structure calculations

The distance restraints were derived from NOESY series spectra and dihedral angle (φ, ψ) restraints were derived from chemical shifts using TALOS (Cornilescu et al., 1999). χ1 angles are determined by comparing cross peak volumes of intra-residual HN-HB and HA-HB NOEs in NOESY spectra with short mixing time. Hydrogen bond restraints were added based on the secondary structure prediction and NOE restraints. The CYANA gave a bad initial structure using restraints from the CANDID module (Herrmann et al., 2002). Therefore we used a model as the initial structure which obtained from homology modeling based on the structure of *E. coli* Grx4 (PDB code 1YKA). Then the initial structure was used as filter model for SANE (Duggan et al., 2001) to obtain the assignments and generate new distance restraints. A new round of CYANA (Guntert et al., 1997) calculation was then run using the new distance restraints. Until the distance restraint violations were smaller than 0.3 Å, 100 structures were selected as the initial structures for refinement using AMBER9 (Pearlman et al., 1995). Finally the 20 lowest energy structures were selected for final analysis. The quality of the final structures was analyzed using MOLMOL (Koradi et al., 1996), and PROCHECK_NMR (Laskowski et al., 1996). The mean structure was

generated using MOLMOL and was energy minimized in AMBER9.

The solution structure of apo GrxS14 has been submitted to PDB with the code 2LKU.

Titration of apo GrxS14 with GSH and K_d determination

A concentration of 0.5 M stock solution of GSH was prepared (pH 7.5). The concentration of apo GrxS14 protein was 0.1 mM, and the NMR sample contained 10 mM DTT in order to keep the reducing condition during the titration. The titration was performed at different [GSH]:[GrxS14] ratios between 0 and 230. 2D ^{1}H-^{15}N HSQC spectra were recorded at each titration point at 25 °C and pH 7.5. NMRView was used to analysis the spectra. The combined chemical shift differences were calculated using the equation (1)

$$\delta = \sqrt{\delta_H^2 + (\delta_{HN}/6.5)^2} \qquad (1)$$

where δ_{H} represents the chemical shift changes of ^{1}H and δ_{HN} represents the chemical shift changes of ^{15}N (Mulder et al., 1999).

Titration of GrxS14 with GSH was performed at 0.1 mM protein (the population of protein in monomeric states is estimated to be ~ 90%), so that the dimerization is negligible in the 2D ^{1}H-^{15}N HSQC experiments. The dissociation constants K_d and δ_0 were determined with non-linear regression by fitting the chemical shifts changes *versus* [GSH]: [GrxS14] ratio to the equation (2)

$$\delta = \delta_0 \frac{(K_d + [L_0] + [P_0]) - \sqrt{(K_d + [L_0] + [P_0])^2 - 4[L_0][P_0]}}{2[P_0]} \qquad (2)$$

where δ is the observed change in chemical shift, δ_0 is the total change in chemical

shift at saturation condition, $[L]_0$ is the total molar concentrations of the apo GrxS14 protein and $[P]_0$ is the total molar concentrations of GSH. Only the residues with significant chemical shift changes were involved in fitting to equation (2) given by Morton *et al* (Morton et al., 1996) from the titration spectra.

HADDDOCK protocol

The docking was performed with HADDOCK 2.0 (Dominguez et al., 2003) software using NMR data as restraint files. The docking model was calculated based on chemical shift changes between different 2D ^{1}H-^{15}N HSQC spectra. First, the combined chemical shift differences were calculated using equation (1). Docking of apo GrxS14/GSH complex was based on the combined chemical shift differences between 0 and 230:1 ([GSH]:[GrxS14]) 2D ^{1}H-^{15}N HSQC titration spectra. Docking of the apo GrxS14 dimer was based on the combined chemical shift changes in 2D ^{1}H-^{15}N HSQC spectra of apo GrxS14 at different concentrations. Residues with combined chemical shift change above the average change value were first selected. Then, the average solvent accessible area for each of those residues was calculated in MOLMOL. Only the residues exhibiting over 50% solvent accessibility were used as active residues. The passive residues were defined as the residues closing to the active residues and showing large (>50%) solvent accessibility. Then AIR restraint files were generated in HADDOCK homepage (http://www.nmr.chem.uu.nl/haddock/) for docking calculation. A total of 1000 rigid-body docking structures were generated with HADDOCK software. The 200 best structures were used for the semi-flexible

simulated annealing and refinement in water. Finally, the reasonable docking structures were extracted through cluster analysis on the final refined structures.

Structure models of apo GrxS14 dimer

Comparison of 2D ^{1}H-^{15}N HSQC spectra of apo GrxS14 without GSH for samples at 0.1 and 0.4 mM, it is found that shifted NH peaks are mainly from residues F35, Q37, K66, W71, G86, D88, I89, V91, E92 and S96 (Fig. S4), which should be involved in the dimerization. For calculating structure models of apo GrxS14 dimer using HADDOCK 2.0, residues F35, Q37, K66, W71, G86, D88, I89, V91, E92 and S96 were defined as active, and residues C33, G34, T38, Q41, Q63, E67, S70, P72, T73, F83, K95, G97 and E98 were defined as passive. The semi-flexible regions were defined by considering all residues within 5Å of another molecule in the top 200 initial rigid-body docking structures. The five pairs of segments C33-Q37, K66-E67, S70-T73, G86-E92 and S96-E98 located at the interface were defined as NCS restraints for keeping the dimer symmetric. Based on the pairwise backbone root-mean-square deviation value, ten best docking structures were chosen from the lowest intermolecular energy cluster of the final round structures for analysis. The intermolecular energy of the lowest cluster was -271.40 ± 66.44 kcal/mol.

Structure models of apo GrxS14/GSH complex

Comparing 2D ^{1}H-^{15}N HSQC spectra of apo GrxS14 without GSH and with 230-fold GSH, it was found that residues with significant combined NH chemical

shift changes (> 0.05 ppm) are distributed on all secondary structure regions. Residues with combined NH chemical shift changes $0.05 < \delta < 0.1$ ppm: T4, T10, K13, K19, Q41, L43, F50, S52, R62, S69, Y78, F83, T90 and E92; $0.1 \leq \delta < 0.2$ ppm: V14, V15, V20, K25, C33, F35, Q37, L56, N58, L61, G64, K66, E67, Y68, W71, F74, Q76, L77, F84, G85, Y94 and Q100; $\delta \geq 0.2$ ppm: S36, V39, Q63, G86, D88, I89 and V91 (Fig. 1D and Fig. S6). Therefore, residues K25, C33, F35, Q37, L56, N58, Q63, K66, E67, W71, G86, D88, I89, V91 and Y94 were defined as active, and residues P31, Q32, G34, T38, Q41, E57, E59, L60, R62, S70, P72, T73, P75, F83, E92, K95 and G97 were defined as passive. The semi-flexible regions were defined by considering all residues within 5Å of another molecule. The CNS topology and topology parameter files of GSH for HADDOCK 2.0 (Dominguez et al., 2003) calculation were derived from the websites http://xray.bmc.uu.se/hicup/ and http://davapc1.bioch.dundee.ac.uk/prodrg/. Based on the pairwise backbone root-mean-square deviation value, ten best docking structures were extracted from the lowest intermolecular energy cluster. The intermolecular energy of the lowest cluster was -158.20 ± 34.55 kcal/mol.

REFERENCES

Cornilescu, G., Delaglio, F., and Bax, A. (1999). Protein backbone angle restraints from searching a database for chemical shift and sequence homology. J Biomol NMR 13, 289-302.

Delaglio, F., Grzesiek, S., Vuister, G.W., Zhu, G., Pfeifer, J., and Bax, A. (1995). NMRPipe: a multidimensional spectral processing system based on UNIX pipes. J Biomol NMR 6, 277-293.

Dominguez, C., Boelens, R., and Bonvin, A.M. (2003). HADDOCK: a protein-protein docking approach based on biochemical or biophysical information. J Am Chem Soc

125, 1731-1737.
Duggan, B.M., Legge, G.B., Dyson, H.J., and Wright, P.E. (2001). SANE (Structure Assisted NOE Evaluation): an automated model-based approach for NOE assignment. J Biomol NMR 19, 321-329.
Ferentz, A.E., and Wagner, G. (2000). NMR spectroscopy: a multifaceted approach to macromolecular structure. Q Rev Biophys 33, 29-65.
Guntert, P., Mumenthaler, C., and Wuthrich, K. (1997). Torsion angle dynamics for NMR structure calculation with the new program DYANA. J Mol Biol 273, 283-298.
Herrmann, T., Guntert, P., and Wuthrich, K. (2002). Protein NMR structure determination with automated NOE assignment using the new software CANDID and the torsion angle dynamics algorithm DYANA. J Mol Biol 319, 209-227.
Johnson, B.A., and Blevins, R.A. (1994). NMR View: A computer program for the visualization and analysis of NMR data. J Biomol NMR 4, 603-614.
Koradi, R., Billeter, M., and Wuthrich, K. (1996). MOLMOL: A program for display and analysis of macromolecular structures. J Mol Graphics 14, 29-32.
Laskowski, R.A., Rullmannn, J.A., MacArthur, M.W., Kaptein, R., and Thornton, J.M. (1996). AQUA and PROCHECK-NMR: programs for checking the quality of protein structures solved by NMR. J Biomol NMR 8, 477-486.
Markley, J.L., Bax, A., Arata, Y., Hilbers, C.W., Kaptein, R., Sykes, B.D., Wright, P.E., and Wuthrich, K. (1998). Recommendations for the presentation of NMR structures of proteins and nucleic acids. IUPAC-IUBMB-IUPAB Inter-Union Task Group on the Standardization of Data Bases of Protein and Nucleic Acid Structures Determined by NMR Spectroscopy. J Biomol NMR 12, 1-23.
Morton, C.J., Pugh, D.J., Brown, E.L., Kahmann, J.D., Renzoni, D.A., and Campbell, I.D. (1996). Solution structure and peptide binding of the SH3 domain from human Fyn. Structure 4, 705-714.
Mulder, F.A., Schipper, D., Bott, R., and Boelens, R. (1999). Altered flexibility in the substrate-binding site of related native and engineered high-alkaline *Bacillus subtilisins*. J Mol Biol 292, 111-123.
Pearlman, D.A., Case, D.A., Caldwell, D.A., Ross, W.R., Cheatham, T.E., III., DeBolt, S., Ferguson, D., Seibel, G., and Kollman, P.A. (1995). AMBER, a package of computer programs for applying molecular mechanics, normal mode analysis, molecular dynamics and free energy calculations to simulate the structural and energetic properties of molecules Comput Phys Commun 91, 1-41.
Schuck, P. (2000). Size-distribution analysis of macromolecules by sedimentation velocity ultracentrifugation and lamm equation modeling. Biophys J 78, 1606-1619.
Wang, L., Ren, X., Li, Y., Rouhier, N., Jacquot, J.P., Jin, C., and Xia, B. (2011). ^{1}H, ^{13}C, and ^{15}N resonance assignments of reduced GrxS14 from *Populus tremula* x *tremuloides*. Biomol NMR Assign 5, 121-124.

Table S1. Restraints and Structure Statistics

NOE restraints	2561
Intra-residue	918
sequential	407
medium-range	168
long-range	211
ambiguous	857
dihedral angle restraints	
φ	56
ψ	52
$\chi 1$	29
hydrogen bond restrains	45
chirality restraints	
omega angle	108
Structure statistics of final 20 conformers	
restraints violations	
distance (> 0.2 Å)	0
dihedral angle (> 5°)	0
rmsd from mean structure (Å)	
secondary-structure backbone atoms	0.47
secondary-structure heavy atoms	1.16
all backbone atoms	1.09
all heavy atoms	1.46
Ramachandran statistics (%)	
most favored regions	89.6
additional allowed regions	6.1
generously allowed regions	1.6
disallowed regions	2.7

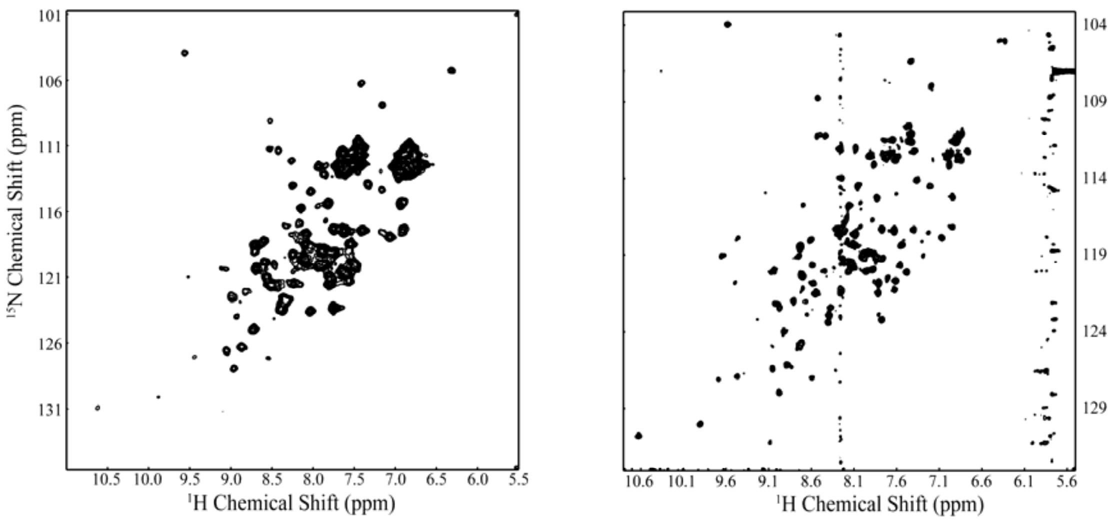

Figure S1. GSH significantly improves the quality of NMR spectra. 2D ^{1}H-^{15}N HSQC spectra of apo GrxS14 at 1.0 mM without (left) and with 20 mM GSH (right), respectively.

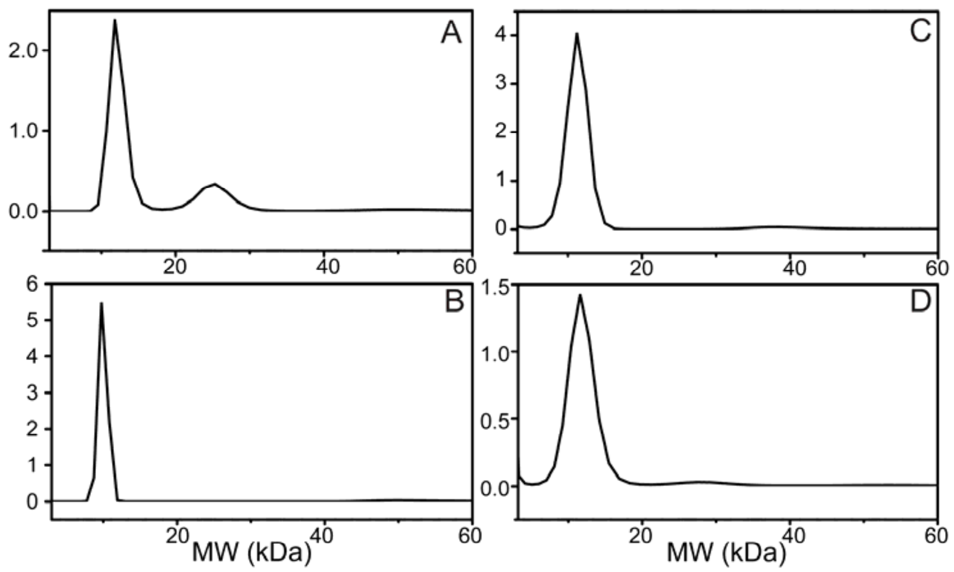

Figure S2. Dimerization of WT and mutant apo GrxS14. Molecular weight determined from analytical ultracentrifuge experiments for WT apo GrxS14 without GSH (A), WT apo GrxS14 with 100 equivalents of GSH (B), F35A mutant of apo GrxS14 (C), and D88A/E92A mutant of apo GrxS14 (D). The sample volume was 400 μL and the protein concentration was ~ 0.075 mM. A wavelength of 280 nm was used to record the UV absorption of the cells which scanned every minute for 5 h. The sedimentation velocity curves were transferred to molecular weight curves using SEDFIT program. The theoretical molecular weight of apo GrxS14 is 12.184 kDa.

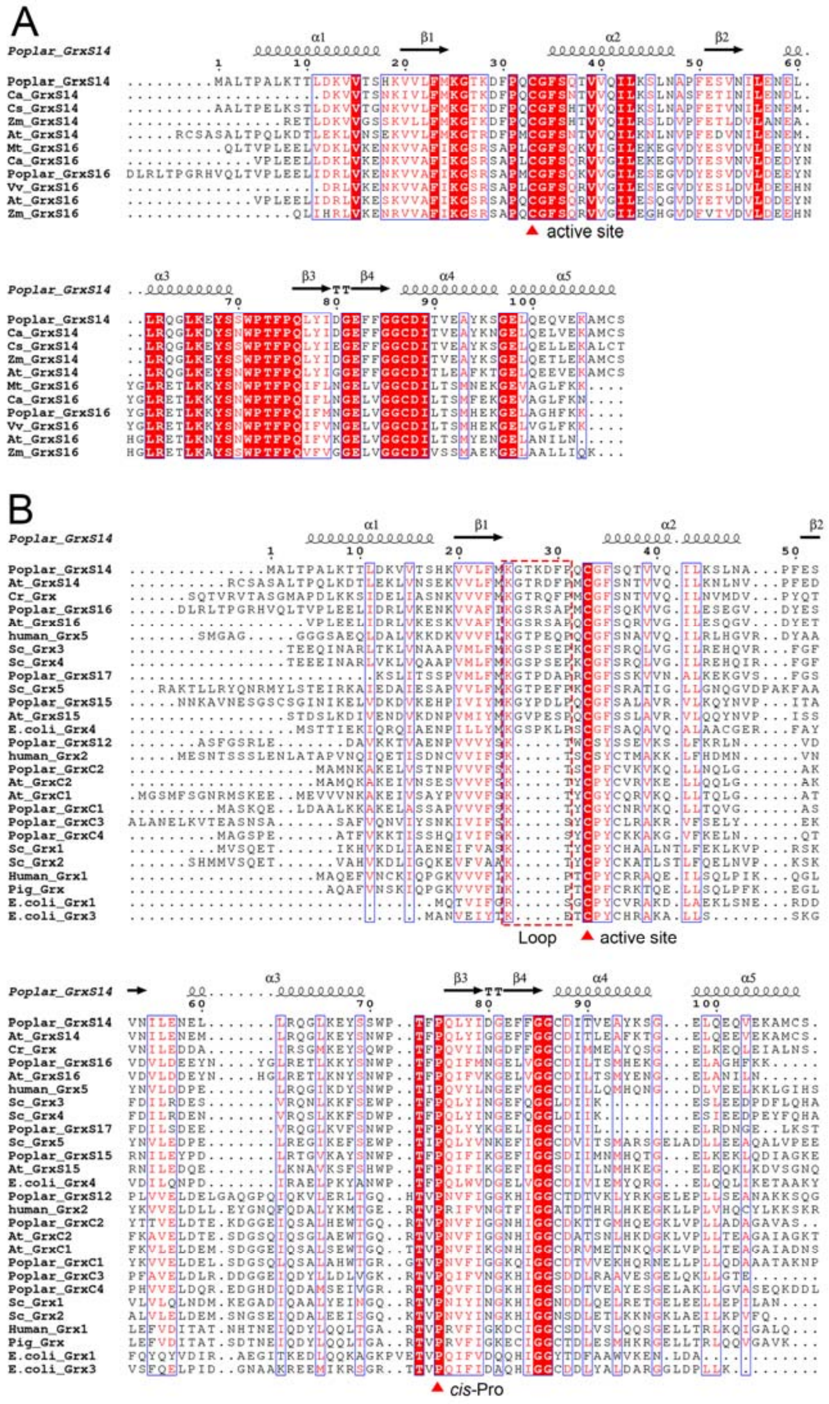

Figure S3. Sequence alignments of Grxs. (A) Sequence alignments of monothiol GrxS14 and GrxS16 in different plants. (B) Sequence alignments of Grxs in different species. Sequences were aligned using ClustalX and represented using ESPript. Secondary structure elements of poplar GrxS14 are shown on the top. The sequence of the loop region between β1 and α2 is indicated as red dashed box. The active site cysteine and conserved *cis*-Pro are labeled on the bottom with red triangles. Poplar: *Populus trichocarpa*, At: *Arabidopsis thaliana*, Cr: *Chlamydomonas reinhardtii*, human: *Homo sapiens*, Sc: *Saccharomyces cerevisiae*, E. coli: *Escherichia coli*, pig: *Sus scrofa*, Ca: *Cicer arietinum*, Cs: *Citrus sinensis*, Zm: *Zea mays*, Vv: *Vitis vinifera*, Mt: *Medicago truncatula*.

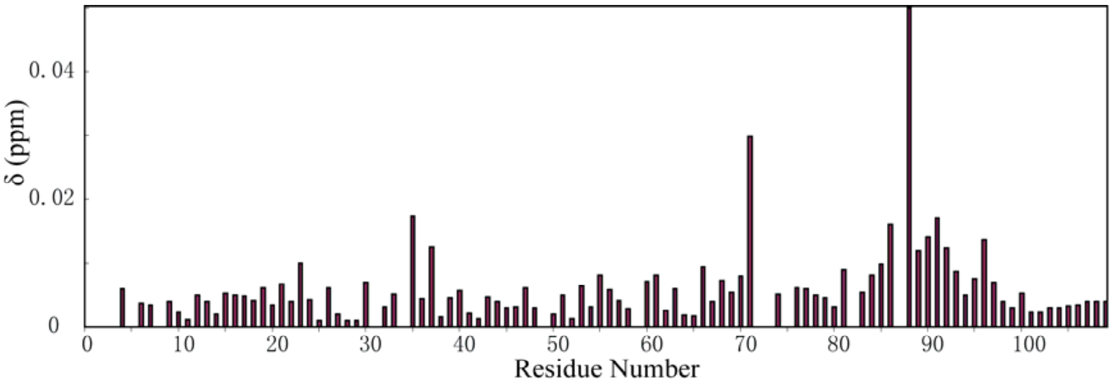

Figure S4. Combined NH chemical shift changes between 0.1 mM and 0.4 mM apo GrxS14. The combined chemical shift changes were calculated using the equation (1). Note that residue 88 is not observed in the spectra of 0.4 mM apo protein.

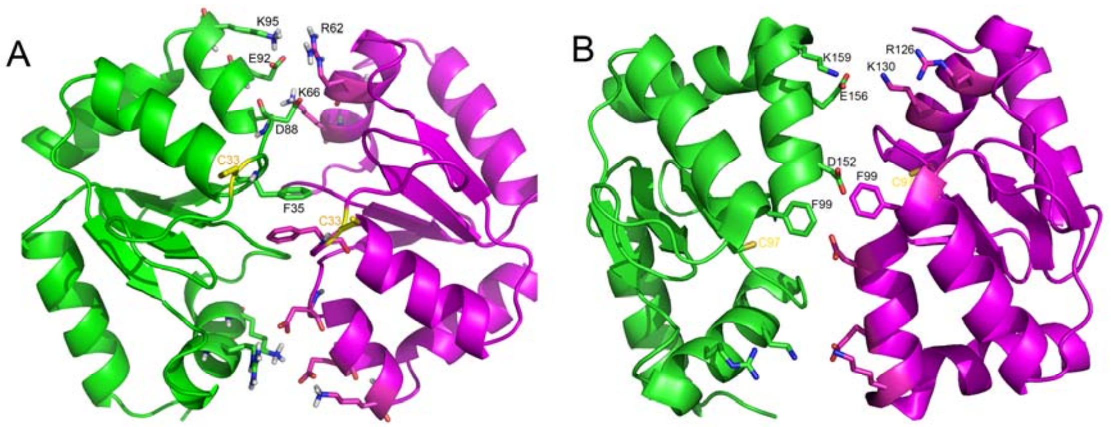

Figure S5. Dimer interface comparison of poplar apo GrxS14 dimer and *Arabidopsis* GrxS14 symmetric dimer. (A) Interface of apo GrxS14 dimer. The active site C33 is shown as yellow. (B) Dimer interface between two molecules with a crystal symmetric operation for the crystal structure of *Arabidopsis* GrxS14 (PDB 3IPZ). For both proteins, side-chains of similar interface residues are shown and labeled.

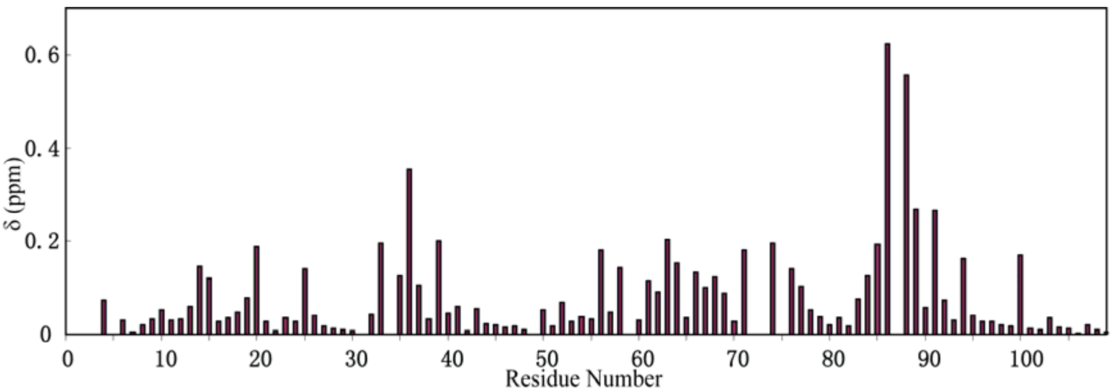

Figure S6. Combined chemical shift changes upon binding of GSH as a function of GrxS14 sequence number. Combined chemical shift changes between free GrxS14 and that with 230 equivalents of GSH are calculated using equation (1).

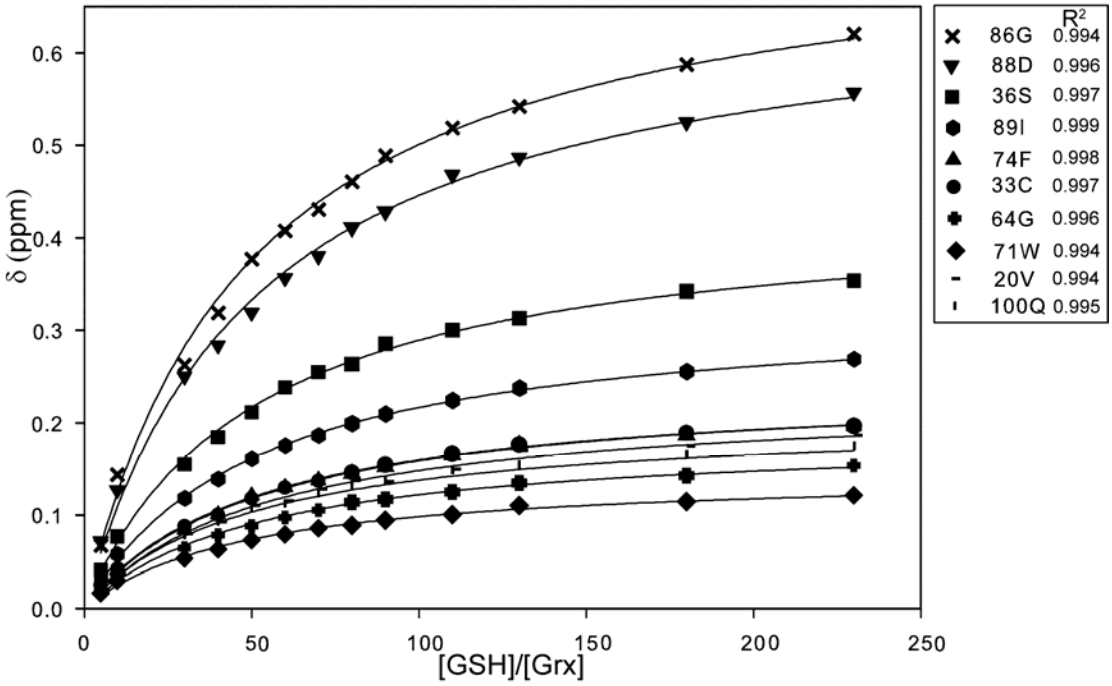

Figure S7. GSH titration curves at 0.1 mM apo GrxS14 as a function of [GSH]:[GrxS14]. The vertical coordinate is the combined chemical shift change with the increase of GSH concentration. Representative residues were chosen to fit the dissociation constant (equation (2)). The K_d values were 4.8±0.3, 5.1±0.3, 5.0±0.2, 5.1±0.2, 5.2±0.2, 5.1±0.2, 5.2±0.3, 4.8±0.3, 5.5±0.3, and 4.9±0.3 for residues G86, D88, S36, I89, F74, C33, G64, W71, V20 and Q100, respectively. The R^2 values using equation 2 fitting the GSH titration curve are also shown.

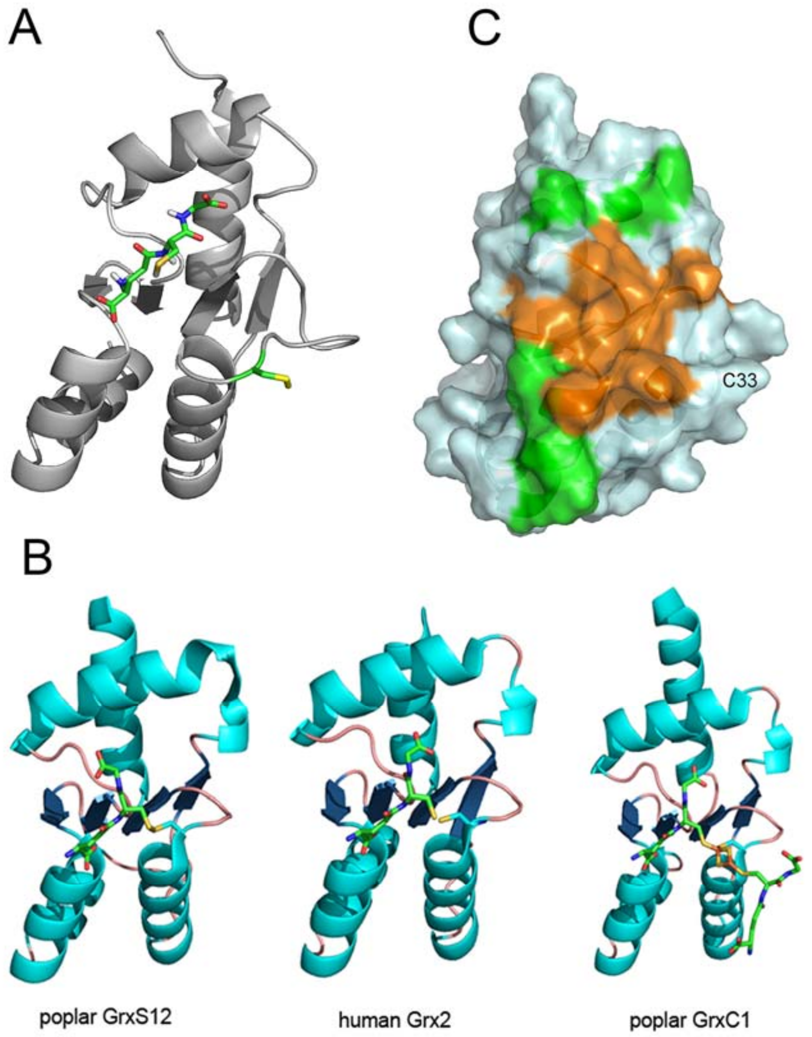

Figure S8. Comparison of GSH binding for different Grxs. (A) GSH binding mode in poplar GrxS14 from GrxS14/GSH complex. GSH molecule is shown as sticks in green and the side-chain of C33 is also shown as stick. (B) GSH binding modes in different structures of Grxs. Poplar GrxS12 (PDB 3FZ9) forms disulfide bond with GSH, human Grx2 (PDB 2FLS) forms non-covalently bond with GSH, and holo poplar GrxC1 (PDB 2E7P) forms [2Fe-2S] cluster with GSHs. GSH molecule is shown as sticks in green. (C) Mapping of the self-association surface and GSH binding site on the GrxS14 solvent-accessible surface. The residues involved only in the self-association are shown as green, and orange indicates the residues involved in both the GSH interaction and dimerization. The active site C33 is also labeled.

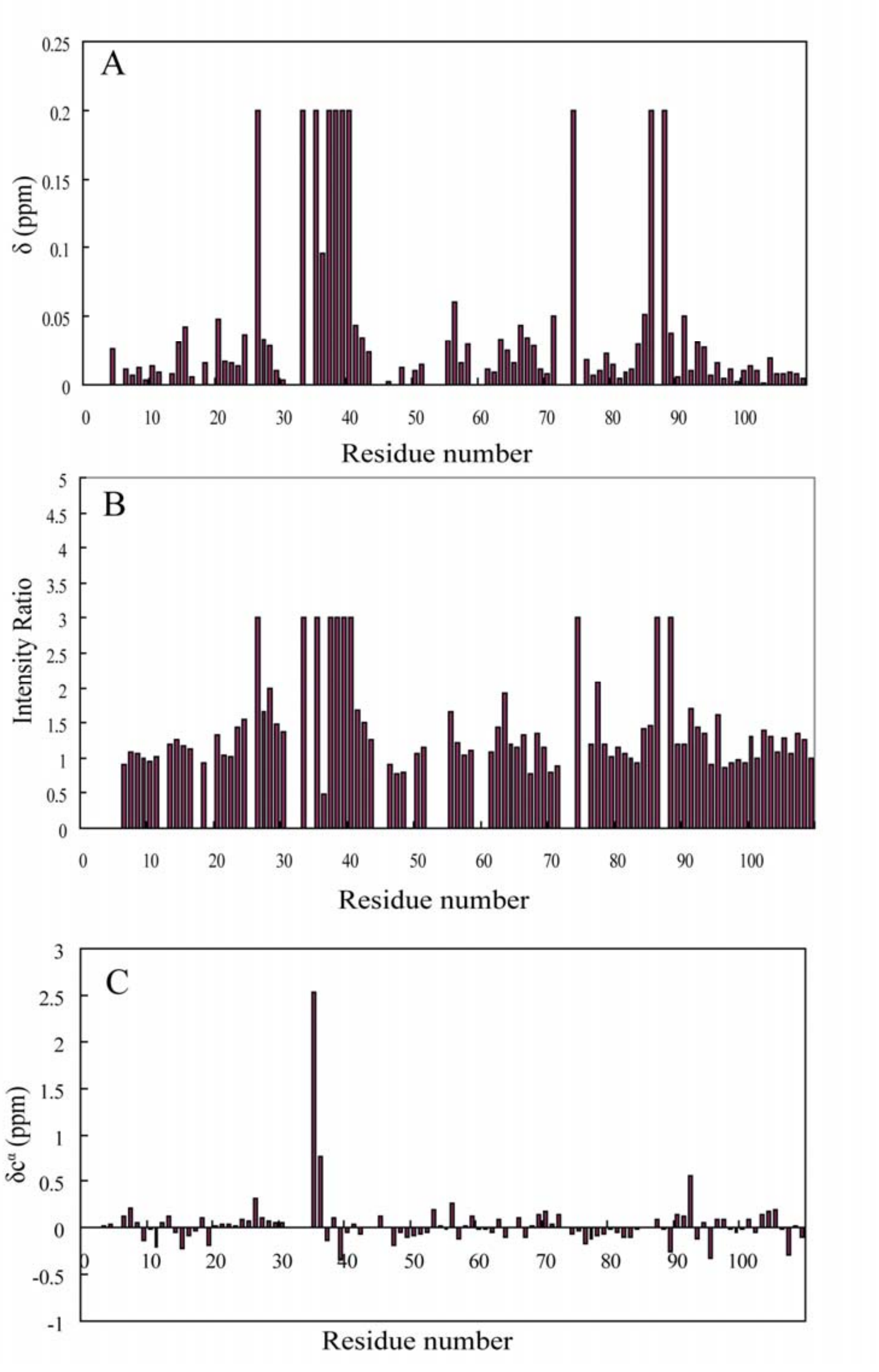

Figure S9. Chemical shift or intensity differences between apo and holo GrxS14. (A) ^{1}H and ^{15}N combined chemical shift differences, δ, vs residue number. The combined chemical shifts between the apo and holo forms GrxS14 were calculated by equation (1). The residues with δ = 0.2 ppm denote the residues not observed in the 2D ^{1}H-^{15}N HSQC spectrum of holo GrxS14. (B) Ratios of normalized intensities of 2D ^{1}H-^{15}N HSQC spectra signals between apo and holo GrxS14 vs residue number. The residues with ratio value 3 denote residues not observed in the HSQC spectrum of holo GrxS14. (C) $^{13}C^{\alpha}$ chemical shift differences between holo and apo GrxS14 vs residue number.
